# Bridging Regenerative and Restorative Stem Cell Therapies in Parkinson’s Disease

**DOI:** 10.3390/cells15070578

**Published:** 2026-03-25

**Authors:** Chiamaka Onuigbo, Juan Martinez-Lemus, Emily Tharp, Mya Schiess

**Affiliations:** Department of Neurology, University of Texas Health Sciences Center at Houston, Houston, TX 77030, USA; chiamaka.c.onuigbo@uth.tmc.edu (C.O.); juan.d.martinezlemus@uth.tmc.edu (J.M.-L.); emily.l.tharp@uth.tmc.edu (E.T.)

**Keywords:** Parkinson’s disease, stem cell therapy, regenerative medicine, restorative therapies, mesenchymal stem cells, neural stem cells, induced pluripotent stem cells, fetal ventral mesencephalic cells, embryonic stem cells, Muse cells

## Abstract

**Highlights:**

**What are the main findings?**

- Stem cell therapies for Parkinson’s disease can be conceptualized along a regenerative-restorative spectrum, determined by cell type and administration route.
- Regenerative approaches (e.g., fetal ventral mesencephalic stem cells, embryonic stem cells, induced pluripotent stem cells) primarily aim to replace lost dopaminergic neurons, while restorative approaches (mesenchymal stem cells) mainly provide neuroprotective and immunomodulatory effects. Some cell types, such as Multilineage-differentiating stress-enduring cells and neural stem cells have both regenerative and restorative features.

**What are the implications of the main findings?**

- Distinguishing regenerative from restorative mechanisms helps explain variability in clinical outcomes across stem cell trials and clarifies the interpretation of therapeutic efficacy.
- This framework can guide rational trial design and exposes the potential for combined or sequential strategies that may yield more durable disease-modifying benefits in Parkinson’s disease.

**Abstract:**

The prevalence of Parkinson’s disease (PD) is projected to rise, stressing the urgency for disease-modifying therapies. Its complex pathophysiology, characterized by α-synuclein aggregation, mitochondrial dysfunction, oxidative stress, and chronic neuroinflammation, continues to complicate therapeutic development. Mounting evidence implicates neuroinflammation as both a driver and consequence of disease progression. This highlights the need to address both neuronal loss and the established dysfunctional microenvironment. Consequently, stem cell-based treatments have generated interest for their immunomodulatory, neuroprotective, and regenerative potential. However, therapeutic outcomes are strongly influenced by stem cell type and route of administration, which together determine whether effects are predominantly regenerative or restorative. In this review, we introduce a conceptual framework that situates stem cell therapies for PD along a regenerative–restorative continuum. Regenerative therapies include fetal ventral mesencephalic, embryonic, and induced pluripotent stem cells. When delivered intracerebrally, they aim to reconstruct dopaminergic circuitry through differentiation and engraftment. In contrast, restorative approaches include mesenchymal stem cells, which exert paracrine and immunomodulatory effects to promote neuroprotection and functional stabilization of the neuronal environment. Multilineage-differentiating stress-enduring cells and neural stem cells exhibit both regenerative and restorative features, to differing extents. This framework integrates mechanistic and clinical evidence that may help clarify distinctions across stem cell approaches and inform future translational development in PD.

## 1. Introduction

Neurodegenerative diseases are predicted to become the second leading cause of death after cardiovascular disease by 2040, according to the World Health Organization (WHO) [1]. As it stands, Parkinson’s disease (PD) affects an estimated 11.77 million people worldwide as of 2021 [2] and its prevalence is expected to increase by 112% by 2050 [3]. While current pharmacological and device-based treatments mainly alleviate symptoms, developing a disease-modifying therapy has proven difficult due to the interplay of chronic neuroinflammation and a multifactorial pathophysiology.

Pathologically, PD is characterized by the destruction of dopaminergic neurons in the substantia nigra pars compacta (SNpc), causing a disruption in dopaminergic neurotransmission. Dopaminergic degeneration triggers oxidative stress and the accumulation of misfolded α-synuclein (α-syn) aggregates, resulting in amplified neuroinflammation. Sustained inflammation creates a favorable environment for α-syn self-propagation, worsening neurodegeneration [4]. Given the interdependent relationship between inflammation and protein aggregation, immunomodulatory and neuroprotective therapies aimed at interrupting the positive feedback loop have garnered attention.

An emerging treatment approach in PD involves the use of stem cell-based therapies either to replace lost nigrostriatal dopaminergic neurons or to restore a permissive host environment that supports neuronal function and repair. Although a wide range of stem cell products and delivery routes are currently being explored, these interventions are often grouped as a single entity despite engaging fundamentally distinct biological mechanisms. A useful distinction between these approaches emerges when both cell type and route of administration are considered.

Because of their capacity to differentiate into dopaminergic neurons and replace damaged cells, fetal ventral mesencephalic (FVM), embryonic (ESC), and induced pluripotent stem cells (iPSC) possess primarily regenerative potential. Alternatively, mesenchymal stem cells (MSC) exert predominantly restorative effects through paracrine activity, fostering an anti-inflammatory environment. Multilineage-differentiating stress-enduring (Muse) cells and neural stem cells (NSC) bridge these categories, displaying both regenerative and restorative properties. However, Muse cells demonstrate more regenerative characteristics, while NSCs exert more functional restorative effects.

**Figure 1:**
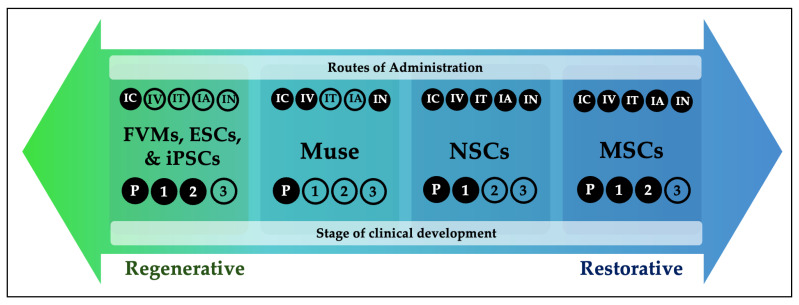
Conceptual regenerative-restorative continuum of stem cell therapies in Parkinson’s disease. Stem cell-based therapies for Parkinson’s disease can be conceptualized along a continuum ranging from predominantly regenerative to predominantly restorative mechanisms. Routes of administration and the stages of clinical development are shown for each cell type. This figure represents a conceptual framework and does not imply superiority or comparative efficacy. Abbreviations: ESCs: embryonic stem cells, FVMs: fetal ventral mesencephalic stem cells, IA: intra-arterial, IC: intracerebral, IN: intranasal, iPSCs: induced pluripotent stem cells, IT: intrathecal, IV: intravenous, MSCs: mesenchymal stem cells, Muse: multilineage stress-enduring cells, NSCs: neural stem cells, P: preclinical phase, 1: phase I clinical trial, 2: phase II clinical trial, 3: phase III clinical trial.

Regenerative effects require successful engraftment and integration into the striatum, typically achieved by bypassing the blood–brain barrier (BBB). On the other hand, restorative effects capitalize on the systemic circulation to modulate immune and trophic signaling to result in neuroprotection and functional restoration. In this review we will introduce a framework that positions stem cell therapies for PD along a regenerative–restorative continuum (Figure 1). This perspective aims to clarify how distinct combinations of cell type and delivery route influence therapeutic outcomes, providing a foundation to guide future therapeutic development.

## 2. Regenerative Therapies

The goal of regenerative medicine is to replace dysfunctional neurons and reestablish neuronal circuitry. This approach can potentially offer superior benefits as it attempts to address the underlying pathology. However, clinical benefit is largely dependent on the ability of the transplanted neurons to reinnervate damaged areas and maintain long-term function. When analyzing studies for regenerative potential, necessary characteristics to define true neuronal replacement include evidence of graft survival in the target region, differentiation into dopaminergic cells confirmed by biomarker expression like tyrosine hydroxylase (TH+) positivity, and integration into the host’s circuitry through synaptic connections [5]. A summary of completed clinical studies evaluating regenerative therapies in PD are presented in Table 1.

### 2.1. Fetal Ventral Mesencephalic Stem Cells

The first experimental intracerebral (IC) transplantation study was performed by Perlow et al. in 1979 [36]. The investigators implanted FVM cells from healthy mice into the lateral ventricles of 6-hydroxydopamine (6-OHDA) lesioned mice. Histochemical analyses revealed extensive dopaminergic proliferation within the grafts, with fibers extending into the caudate. Behavioral improvement was observed primarily in mice with complete SNpc denervation and in those with more anterior graft placement. This robust response was attributed to a combination of denervation-induced dopamine receptor super-sensitivity, lack of competing dopaminergic terminals, and increased access to extracellular matrix cues, all of which facilitated host to graft integration. Furthermore, anterior placement within the striatum, as opposed to the SNpc, preferentially promoted differentiation into A9 dopaminergic neurons [37], which is a subtype that is associated with motor control in PD [38]. Wuerthele et al. confirmed that FVM-derived neurons displayed electrophysiological and pharmacological properties nearly identical to native SNpc neurons [39]. Subsequent preclinical studies in animal models consistently demonstrated FVM graft survival, dopaminergic differentiation, functional neurotransmitter release, and partial reconstruction of host circuitry [40,41]. Collectively, these findings across multiple parkinsonian models provided the scientific rationale to initiate clinical trials in humans.

In 1988, Lindvall et al. conducted the first human FVM cell transplantation, resulting in sustained symptomatic improvement beginning 2 months post-transplantation [6,28,29]. PET imaging demonstrated a 130% increase in tracer uptake within the grafted striatum at 5 months, signaling increased dopamine synthesis and storage. In 2001, Freed et al. performed a double-blinded, sham surgery-controlled study to implant FVM cells into patients with advanced PD [20]. Despite similar findings of increased dopaminergic uptake on PET imaging and post-mortem confirmation of robust dopaminergic fiber outgrowth throughout the forebrain, the trial did not reach statistical significance for motor outcomes between the grafted and sham surgery groups. Similarly, Olanow et al. investigated the same paradigm in a larger cohort and reported comparable results [22]. These findings contrasted earlier, non-blinded, smaller studies which showed clinical benefit, albeit to varying degrees [8,9,10,11,12,13,14,15,16,17,18,19,20,22,34,35]. Participants in these studies also experienced graft-induced dyskinesias (GIDs), which were not responsive to reductions in levodopa and in some cases resulted in substantial disability [10,15,42]. More recently, the TRANSEURO trial aimed to evaluate FVM grafts under more stringent conditions, including improved cell-sorting practices to reduce GIDs, standardized surgical procedures, and protocols producing consistent, viable grafts [24]. Despite these refinements, TRANSEURO failed to show a substantial clinical benefit, highlighting the limitations of FVM-derived graft transplantation in PD.

### 2.2. Human Pluripotent Stem Cells

Due to the limited donor availability, variable clinical effects, and ethical concerns surrounding the use of FVM tissue [43], the field transitioned to exploring human pluripotent stem cells (hPSCs), like ESCs or iPSCs. ESCs are pluripotent cells derived from the inner cell mass of a blastocyst, with the ability to differentiate into any type of cell in the body [44]. With this cell type, there is risk of teratoma formation, difficulties with cell regulation, and the need for immunosuppression. IPSCs are adult somatic cells (e.g., fibroblasts) that are genetically reprogrammed to an embryonic-like pluripotent state [45]. While iPSCs and ESCs share functional similarities, including equivalent lineage specification capabilities [46], they are not identical. IPSCs offer the advantage of autologous use in idiopathic PD, thereby reducing the need for immunosuppression. However, reprogramming may amplify preexisting genetic mutations within the donor line, increasing the risk of tumorigenicity [47]. Therefore, in individuals with known genetic forms of PD, autologous-based iPSC therapies may require additional screening or gene-correction strategies before clinical use [48].

#### 2.2.1. Embryonic Stem Cells

Deacon et al. were the first to demonstrate that ESC-derived grafts are capable of differentiating into a dopaminergic phenotype after transplantation, without requiring in vitro pre-treatment with patterning growth factors [49]. However, early ESC transplantation studies showed inconsistent results. While grafted cells were viable and could proliferate, functional benefits varied [50]. This variation was largely attributed to conventional differentiation methods producing mixed cell populations, resulting in grafts composed of GABAergic and serotonergic cells alongside dopaminergic cells [51,52].

To reduce teratoma and GID risk, Takagi et al. implemented early differentiation strategies that improved survival and phenotypic stability of ESC-derived dopaminergic neurons in 6-OHDA treated rat models [51]. These strategies improved safety but failed to produce regionally specific A9 dopaminergic neurons. Subsequent advances in differentiation paradigms enabled the production of more homogenous populations of A9 dopaminergic neurons [53,54]. These newer protocols demonstrated similar preclinical efficacy to FVM grafts in rodent PD models, yet with a substantially reduced side effect profile [55]. Informed by these findings, Kim et al. further optimized progenitor maturation and regional identity markers, resulting in enhanced scalability, safety, and therapeutic potency [56].

To date, only 4 ESC-derived dopaminergic neuron transplantation studies have been conducted or are planned in humans. Two phase I/IIa dose-escalation trials in North America and Asia demonstrated safety of these products, as well as dose-dependent increases in dopamine-specific tracer uptake on PET imaging [25,27]. Exploratory clinical outcomes also exhibited stabilization or improvement of motor scores in a dose-dependent manner. Currently, another phase I/IIa dose escalation trial in Europe (STEM-PD), is underway, with early results indicating a favorable safety profile and clinical benefit in the lower dose cohort [57]. In addition, BlueRock Therapeutics is recruiting for a phase III trial, using their ESC-derived product, bemdaneprocel, to evaluate long-term efficacy and safety outcomes in a larger population of patients with PD [58].

#### 2.2.2. Induced Pluripotent Stem Cells

IPSCs utilize differentiation protocols and developmental signaling pathways comparable to those used for ESCs, requiring similar maturation steps to generate A9 neurons [59]. Advances in these protocols, including improved lineage specification and cell-sorting techniques, have substantially reduced the risk of tumorgenicity, as reflected by low Ki-67 expression in differentiated cell populations. To date, no teratoma formation has been reported in clinical studies using iPSC-based therapies. This improved safety profile is reinforced by the utilization of non-integrating or excisable programming methods, shortened culture times, synthetic mRNA approaches, and genome-editing strategies such as CRISPR to reduce oncogenic risk [47]. Although modern differentiation protocols also address issues, such as residual epigenetic memory and donor genetic background variability, to produce more consistent dopaminergic neuron yields [60,61], differentiation efficiency and scalability remain suboptimal for large-scale clinical translation, limiting their clinical utility [47].

Preclinical models, which tend to generate iPSC lines from the same donor, consistently demonstrate that transplanted cells integrate into host’s circuitry, leading to improvement in functional imaging and overall behavior [62,63]. Additionally, microglial density surrounding the graft was not statistically different from that seen with sham surgery, indicating minimal immune system activation and the feasibility of transplantation without immunosuppression [62]. Despite the manufacturing challenges, the combination of comparable functional outcomes in preclinical studies, reduced immunogenicity, and fewer ethical constraints positions iPSC-derived products as a more clinically viable and promising regenerative cell source than ESCs.

In 2018, the first human transplantation of iPSC-derived dopaminergic progenitors was conducted by the Takahashi group in Kyoto, Japan [64]. Their phase I/IIa trial demonstrated safety and displayed dose-dependent improvements in motor performance and dopamine synthesis on PET imaging over 24 months [26]. Additionally, a case report by Schweitzer et al. described sequential, bilateral iPSC graft implantation, showing modest increases in PET tracer uptake over 24 months that correlated with motor improvement and stabilization [23]. More recently, The Neuroregeneration Research Institute (McLean Hospital) initiated a Phase I feasibility and safety trial of autologous iPSC-derived grafts for PD at Brigham and Women’s Hospital in the United States. Preliminary results of this trial are pending [65].

### 2.3. Alternative Administration Routes

To date, all reported studies using FVM cells, ESCs, and iPSCs have employed IC transplantation as the route of administration. This approach is preferred because direct integration into the striatum or midbrain is necessary for dopamine reinnervation and functional neuronal replacement. These cells rely on regional cues from the surrounding glial and neuronal cells present in the striatum or SNpc to properly differentiate into dopaminergic cells [66]. Moreover, their migratory capacity is limited to only a few millimeters [67]. These benefits would be lost if delivered systemically, as the cells lack active homing or chemotactic migration signals to traverse long distances to the brain [68] and cannot cross the BBB [68]. Ultimately, although FVM cells, ESCs, and iPSCs possess intrinsic regenerative potential, administration via routes other than IC injection substantially diminishes their capacity to reconstruct dopaminergic circuitry.

## 3. Restorative Therapies

The goal of restorative medicine is to enhance or restore the body’s endogenous capacity to heal and maintain optimal function. This approach offers an advantage by attempting to revert damage driven by ongoing inflammation through the reinforcement of innate repair pathways. When considering restorative therapies in PD, there is greater flexibility in the choice of administration route, as direct penetrance of the BBB is usually not required. Notably, studies aimed at restorative potential also report neuroprotective effects, such as increased TH+ expression without evidence of stem cell differentiation into dopaminergic cells or integration into the host’s central nervous system. A summary of completed clinical studies evaluating restorative therapies in PD is presented in Table 2.

### 3.1. Mesenchymal Stem Cells

MSCs are multipotent stromal cells derived from a variety of adult and perinatal tissues, with the capability of self-renewal and differentiation into multiple cell lineages. An advantage of MSC-based therapies include safety with allogeneic use, which improves its scalability and procurement in relatively short timeframes. Early concerns regarding allogenic transplantation centered on the risk of immune rejection and the presumed need for immunosuppression. However, accumulating clinical evidence across multiple diseases has revealed the safety of MSC administration even in the absence of immunosuppression, largely due to their low expression of class II HLA molecules and immunomodulatory properties [69]. Despite its favorable safety profile, HLA mismatch, repeated dosing, and inflammatory conditions can increase the risk of immune responses, highlighting the need for continued immunological monitoring.

Common tissue sources include muscle, skin, adipose tissue, umbilical cord blood, and bone marrow, among others. In the PD literature, the most frequently studied sources are bone marrow (BM-MSC), adipose tissue (Ad-MSC), and umbilical cord-derived MSCs (UC-MSC), with each offering slightly distinct advantages. Despite similar surface antigen expression profiles and immunosuppressive properties among MSC sources, Ad-MSCs can be harvested in the highest quantities per collection, allowing for the shortest culture time to reach a therapeutically relevant dose [70]. These cells also have a relatively high basal secretion of neurotrophic factors, although their lower intrinsic proliferation rate may limit extensive population expansion [71,72]. UC-MSCs display the lowest expression of senescence markers, resulting in the highest proliferative capacity and preservation of their intrinsic anti-inflammatory properties [72]. Finally, BM-MSCs secrete higher levels of VEGF and TGF-β, as well as display superior homing capabilities, allowing for more robust systemic effects [72].

In the 1970s, Friedenstein et al. first discovered fibroblast-like cells isolated from bone marrow, now recognized as MSCs, that demonstrated the ability to form bone after being cultured under specific inducive factors [73]. As evidence accumulated supporting their therapeutic potential across various disease states, MSCs gained attention as a possible treatment for neurodegenerative disorders. In 2001, Li et al. demonstrated the clinical benefit of MSCs in a PD mouse model [74]. This new cell population was popularized as a therapeutic option due to its accessibility from various tissue sources, avoidance of ethical concerns, immunoprivileged status, and relatively high genetic stability [75,76]. Additionally, MSCs can migrate to damaged areas through a network of chemotactic signaling, most notably the stromal-cell derived factor-1α (SDF-1α)/CXCR4 axis, and cell adhesion molecules, facilitating the investigation of multiple routes of administration [77,78].

**Table 2:** List of Completed Clinical Studies in Parkinson’s Disease Using Restorative Therapies.

First Author (Year)	Stem Cell Type	Delivery Route	Study Design	Sample Size	Follow-Up (Months)	Age ^a^	Disease Duration (Years) ^a^	Clinical Outcomes	Safety/Adverse Events
**Brazzini et al. (2010)** [79]	autologous BM-MSCs	IA	phase I	53	12	62.5 ± 10.4	9.3	Improvement in UPDRS, QoL, and ADL scores	Psychosis, common colds
**Venkataramana et al. (2010)** [80]	autologous BM-MSCs	IC	phase I	7	10–36	55.4 ± 15.4	14.7 ± 7.56	Improvement in UPDRS III, ADL scores seen in 3 patients	NR
**Yun et al. (2011)** [81]	UC-MSCs	IA	case series	8	1	58.4 ± 9.3	10 ± 6.9	Improvement in UPDRS and ADL scores	Fatigue, fever, euphoria
**Venkataramana et al. (2012)** [82]	allogeneic BM-MSCs	IC	phase I	12 (8 PwPD)	12	58.8 ± 10.2	7.75 ± 3.5	Improvement in UPDRS in PwPD, early disease showed more improvement	NR
**Aili et al. (2013)** [83]	UC-MSCs	IT	phase I	9	6	67.1 ± 5.3	NR	Improvement in UPDRS	fever
**Dapeng et al. (2013)** [84]	UC-MSCs	IT	phase I	30	3	56	NR	Improvement in UPDRS total, II, and III scores	NR
**Xiaoquin et al. (2013)** [85]	NSC	IT	phase I	22	2	62 ± 4.2	NR	Improvement in UPDRS	NR
**Lige et al. (2014)** [86]	NSCs	IV	phase I	21	24	57.3 ± 9.1	NR	Improvement in UPDRS, H&Y and QoL measures	NR
**Yan et al. (2014)** [87]	UC-MSCs	IA	case series	15	1	63.4 ± 7.9	NR	Improvement in UPDRS	Fever, fatigue
**Boika et al. (2020)** [88]	autologous BM-MSCs	IN, IV	pilot study	23	3	52 (39.5, 59) intervention; 52 (47.5, 62.5) placebo	7 (5, 8) intervention; 6 (4, 7) placebo	Improvement in MDS-UPDRS and nonmotor scores, reduced OFF time	NR
**Schiess et al. (2021)** [89]	allogeneic BM-MSCs	IV	phase I	20	12	66	4–6	Dose dependent improvement in UPDRS total, III scores, and H&Y in OFF state	No serious AEs related to the product; most AEs were mild and transient
**Shigematsu et al. (2021)** [90]	autologous Ad-MSCs	IV	case series	3	6	75 ± 4.4	9.7 ± 6.4	Gradual improvement in UPDRS I—III that plateaued after 3rd infusion	NR
**Vij et al. (2023)** [91]	autologous Ad-MSCs	IV	case report	1	30	77	>17 years	Improvement in UPDRS, decrease in dyskinesias and LEDD	NR
**Jiang et al. (2024)** [92]	NSCs	IN	phase I	18	12	61.8 ± 7.6	10.7 ± 3.9	Non-dose-dependent improvement in MDS-UPDRS total and II, III score; improvement in H&Y and LEDD; no changes in non-motor symptoms	No significant difference in severity of AE between groups; non-serious AEs were mild and transient
**Schiess et al. (2025)** [93]	allogeneic BM-MSCs	IV	phase II	45	22	66.5	3	3 infusion arm improved MDS-UPDRS more than 2 infusion arm and placebo; improvement in QoL measures in treatment arms	AEs were mild and transient; no severe AEs; general malaise, flu-like symptoms, vomiting, headache
**Vij et al. (2025)** [94]	autologous Ad-MSCs	IV	phase I	10	6	79.4 ± 3.95	NR	No statistical improvement in MDS-UPDRS; significant improvement in PDQ-39	Most AEs were mild; none of the severe AEs were due to the product; flu-like symptoms, fatigue

Abbreviations: **ADL**: activities of daily living; **AE**: adverse event; **Ad-MSC**: adipose tissue mesenchymal stem cell; **BM-MSC**: bone marrow mesenchymal stem cell; **H&Y**: Hoehn and Yahr scale; **IA**: intraarterial; **IC**: intracerebral; **IN**: intranasal; **IT**: intrathecal; **IV**: intravenous; **LEDD**: levodopa equivalent daily dosing; **MDS-UPDRS**: Movement Disorder Society—United Parkinson’s Disease Rating Scale; **NR**: not reported; **NSC**: neural stem cells; **PDQ-39**: Parkinson’s Disease Questionnaire-39; **PwPD**: patients with Parkinson’s disease; **QoL**: quality of life; **UC-MSC**: umbilical cord mesenchymal stem cell; **UPDRS**: United Parkinson’s Disease Rating Scale. ^a^ The values are given as mean ± SD or median (Q1, Q3), depending on the information that is given in the articles.

Early researchers hypothesized that this cell type might possess regenerative properties similar to FVM cells, ESCs, or iPSCs. Initial in vitro studies showed that MSCs could be induced to adopt a dopaminergic-like phenotype with the use of growth factors, as evidenced by increased TH+ expression [95,96]. However, despite optimized differentiation conditions, these cells failed to express key dopaminergic markers and lacked the electrophysiological properties that are consistent with mature, functional dopaminergic neurons [97]. Furthermore, in vivo studies lacked definitive evidence of the generation of newly formed dopaminergic cells following transplantation, weakening the hypothesis that MSCs improve symptoms through regeneration [98,99]. Additionally, despite the capacity of MSCs to migrate to sites of injury, only a small proportion of cells reached target regions within the brain. Collectively, these findings redirected the hypothesis of a cell-replacement mechanism to a neuroprotective mechanism in which MSCs primarily promote the survival and function of existing dopaminergic neurons via indirect means [100].

### 3.2. Mechanism of Action

MSCs utilize systemic and paracrine methods that modulate immune and neural function. Primarily, MSCs offer neuroprotection by secreting bioactive factors that collectively comprise the MSC secretome. This secretome includes a diverse array of growth factors, cytokines, chemokines, extracellular vesicles, and microRNAs that coordinate signaling pathways involved in immune repair and regulation. MSCs have been shown to release multiple neurotrophic factors such as GDNF, BDNF, NGF, IGF, and VEGF, which stimulate the endogenous repair system by enhancing synaptic plasticity and strengthening surviving circuits [101]. VEGF also contributes to controlled angiogenesis and facilitates the restoration of the BBB integrity, limiting systemic inflammatory signals that exacerbate neuroinflammation [100]. Additionally, MSCs contain microRNAs that can regulate neurogenesis-related pathways and can reprogram host cell responses, creating a reparative environment amid ongoing inflammation [102].

In addition to their neuroprotective effects, MSCs manipulate both innate and adaptive immune cells through multiple mechanisms. For example, after engulfment by antigen-presenting cells, MSC antigens are processed and presented in a manner that promotes a tolerogenic immune response, driving activated microglia toward an anti-inflammatory phenotype [103]. MSCs also can inhibit T-cell activation and proliferation through the release of immunoregulatory factors such as TGF-β1 [100] and can suppress B-cell proliferation by limiting differentiation and chemotaxis [104]. Collectively, these mechanisms promote the upregulation of anti-inflammatory cytokines such as IL-6, IL-10, and TGF-β, while simultaneously downregulating pro-inflammatory cytokines like TNF-α, thereby reshaping the immune environment [105].

MSCs can also mitigate oxidative stress, a key contributor to dopaminergic neurodegeneration in PD. Oxidative stress arises when the body’s endogenous antioxidant defenses are insufficient in clearing reactive oxygen species (ROS) in the brain, which preferentially damage areas like the SNpc [106]. In PD, deficiencies in mitochondrial complex I activity contribute to impaired mitochondrial function, further exacerbating oxidative stress [107]. Studies indicate that MSCs can reduce oxidative damage, as evidenced by the reduction in malondialdehyde, a marker of lipid peroxidation, and nitrous oxide levels [108]. MSCs can also improve mitochondrial function by repairing damaged mitochondria and increasing mitochondrial complex I activity, thereby reducing free radical production [109]. Additionally, MSCs release antioxidant enzymes, including glutathione peroxidase and superoxide dismutase [110], further contributing to the protection of vulnerable neuronal populations.

A final key mechanism by which MSCs offer neuroprotection is through the suppression of apoptosis responsible for progressive dopaminergic neuron loss in PD. This effect is mediated in part through the release of anti-apoptotic neurotrophic factors, such as IGF-1, HGF, BDNF, and GDNF, and cytokines like IL-10 and TGF-β. SDF-1α, a chemokine secreted by MSCs, exerts an anti-apoptotic effect in cells exposed to toxic environments, while promoting dopamine release [111]. At the intracellular level, MSCs up-regulate anti-apoptotic proteins like Bcl-2, and down-regulate pro-apoptotic proteins and enzymes like Bax and caspase-3 [112]. Emerging evidence from PD models also demonstrates that MSCs can transfer functional mitochondria to damaged dopaminergic neurons, reducing apoptotic signaling and promoting neuronal survival [110].

### 3.3. Evaluation of Administration Routes

Reflecting the initial hypothesis that MSCs had regenerative capabilities, early studies investigated direct IC transplantation, an approach that progressed to human trials after multiple preclinical models consistently demonstrated therapeutic efficacy [113]. Two pilot studies initiated by Venkataramana et al. investigated the safety and efficacy of unilateral and bilateral MSC transplantation [80,82]. Patients with PD were followed for 12 months, during which improvements in Unified Parkinson’s Disease Rating Scale (UPDRS) scores were observed, with greater benefits noted in those with a shorter disease duration. Informal follow-up extending to 36 months revealed no obvious clinical worsening. Although these studies demonstrated the safety and feasibility of IC delivery, the predominantly paracrine mechanism of MSCs prompted increased interest in less invasive approaches.

Owing to the relative ease of administration, most clinical trials investigating MSCs in PD have employed IV delivery. While numerous preclinical PD models have evaluated MSC IV administration, only a limited number of trials have reported detailed dose–response relationships following IV administration in PD. Schiess et al. reported a phase I dose-escalation study evaluating IV allogeneic BM-MSCs, showing that doses up to 10 × 10^6^ cells/kg were safe, well tolerated, and non-immunogenic. Exploratory analyses suggested dose-related motor improvement, reductions in peripheral inflammatory markers, increased BDNF levels, and increased basal ganglia perfusion. Although these effects appeared to wane by 12 weeks, a significant reduction in UPDRS motor scores from baseline to 52 weeks was sustained in the group receiving 10 × 10^6^ cells/kg [89]. Based on these findings, a subsequent phase II randomized, placebo-controlled trial examined repeated IV administration of BM-MSCs and found that three infusions were associated with a higher probability of achieving clinically meaningful improvement in OFF-medication motor scores compared with placebo [93]. Pending analyses of imaging and fluid biomarkers are expected to further clarify the restorative mechanisms underlying these clinical effects.

In parallel with IV approaches, other delivery methods have been investigated in an effort to improve MSC delivery to the central nervous system, including intra-arterial (IA) [81,87], intranasal (IN), and intrathecal (IT) routes. In preclinical studies, Cierri et al. investigated intracarotid BM-MSC infusions and noted that permeation across the BBB was minimal without the use of mannitol to transiently disrupt the barrier [114]. Following the infusion, the MSCs spread to both hemispheres, yet no significant clinical improvements were detected. Of note, the striatal neurons exhibited restored responsiveness to dopaminergic stimulation, as evidenced by normalization of apomorphine-induced rotational behavior. These findings suggest that MSCs may modulate dopaminergic circuitry function even in the absence of neuronal replacement with this method, highlighting potential utility after further optimization. To date, only one clinical trial has employed this route in PD, using autologous BM-MSCs in 53 patients to evaluate safety [79]. No major complications were noted, and over half of the patients achieved at least a 50% improvement in UPDRS scores, with peak effects emerging as early as 1 week in some cases, and after 1 to 3 months in others. Despite these findings, further investigation to evaluate the long-term efficacy and safety of IA delivery is necessary, particularly pertaining to the risk of subclinical ischemic lesions from micro-occlusions that were detected on follow up imaging [115,116].

Among these alternative routes, IN administration has attracted particular interest due to its ability to reach deep brain regions without requiring direct passage across the BBB. Danielyan et al. first evaluated this approach in PD rat models, reporting that approximately 24% of administered BM-MSCs reached the brain and survived for up to 4.5 months, corresponding with sustained behavioral improvements [117]. Unfortunately, a large number of MSCs were detected in the stomach, indicating that a considerable amount of MSCs were swallowed during administration. Subsequent studies similarly observed clinical improvements with this route, though migration efficiency remained low [118]. Translation of these results to humans may prove difficult as migration distances are substantially greater than that in mice. Due to the immunocompetent nature of the nasal mucosa, there is a theoretical concern regarding immune rejection of allogeneic cells. Despite being immunologically active, multiple small scale preclinical and clinical studies have demonstrated that IN-delivered allogeneic MSCs are feasible and safe, even with repeated dosing or with long-term follow-up [119,120,121]. In addition, IN-delivered substances preferentially migrate along CNS-associated pathways rather than within peripheral immune compartments, further reducing the risk of immune activation [122].

IT administration is an attractive administration route, as it circumvents the BBB and allows for more widespread delivery of MSCs and their growth factors to the brain without requiring invasive surgery [123]. Aili reported clinical improvement 28 days after IT administration of MSCs, which was sustained at 6 months [83]. Dapeng et al. conducted a randomized trial, utilizing 4 IT MSC injections, each spaced 1 week apart, resulting in lower UPDRS scores at the end of the study [84]. IT administration was found to be safe, yet it was associated with a higher incidence of adverse events compared to IV delivery [124]. Furthermore, the efficacy of this route may be limited by CSF filtration that reduces cell delivery and the potential risk of hydrocephalus following stem cell administration [123,125].

Given the variety of administration routes, a key question remains regarding which approach is most effective. While individual studies have suggested the superiority of IC over IV administration from an efficacy standpoint [108], multiple meta-analyses indicate that IV, IC, and IT infusions can all produce beneficial effects, with IV administration potentially offering greater efficacy than IC [113,126]. It should be noted that these metanalyses did not include IA or IN studies in their comparisons. Moreover, these conclusions should be interpreted with caution given the paucity of completed, blinded MSC trials in humans with PD.

## 4. Dual-Effect Cell Populations

While the previously discussed stem cell types primarily exhibited either regenerative or restorative potential in preclinical models, other stem cell populations demonstrate a combination of both in neurological disease models, notably Muse cells and NSCs. Muse cells function primarily as regenerative, whereas NSCs are primarily restorative, although both cell types also exhibit secondary functions characteristic of the other category. Muse cells are a subpopulation of MSCs [127], while NSCs are found primarily in the subventricular zone of the lateral ventricles and the hippocampal dentate gyrus [128]. Muse cells are pluripotent like ESCs or iPSCs, whereas NSCs are considered multipotent, as their differentiation is restricted to major neural lineages [129]. Given the potential to simultaneously provide dopaminergic replacement and immunomodulation, these dual-effect cell populations are of increasing interest to investigators as they may offer an avenue towards improved clinical outcomes [130].

### 4.1. Multilineage-Differentiating Stress Enduring Cells

While there are few studies looking at the use of Muse cells specifically in PD models, the effects of Muse cells within the brain have been evaluated in other neurological disease models. Shimamura et al. demonstrated the regenerative potential of intracerebrally transplanted Muse cells in an intracerebral hemorrhage (ICH) mouse model [131]. Compared to control and non-Muse MSC cohorts, mice treated with Muse cells exhibited better functional recovery after the insult. Histological analyses revealed spontaneous differentiation into neuronal phenotypes, axonal sprouting in the subcortex, and structural integration within host neural tissue. Similar findings have been reported in ischemic stroke models [132,133,134], further supporting the regenerative capability of Muse cells. Behavioral and functional benefits have also been reported in spinal cord injury, stroke, and hypoxic ischemic encephalopathy (HIE) utilizing IV administration of Muse cells [135,136,137]. In these studies, Muse cells selectively migrate to injured regions via sphingosine-1-phosphate receptor mediated signaling and localize within the affected tissue. Expression of mature neuronal markers, including NeuN or MAP2 [135,138], together with region-specific migration and functional improvement, supports functional incorporation of Muse cells within injured neural regions.

Although less explored, the ability of Muse cells to restore function in neurodegenerative conditions has shown early promise. Yamashita et al. revealed that IV administration of Muse cells improved motor performance and preserved motor neuron and myofiber integrity compared to controls in an ALS model [139]. Histological examination showed differentiated Muse cells in the pia mater and ventral horn of the spinal cord, suggesting localized engraftment and neural differentiation. Furthermore, Aprile et al. created differentiation protocols that successfully generated GABAergic, glutaminergic, dopaminergic, and astrocytic phenotypes from Muse cells [140]. About 78% of differentiated cells expressed mature neuronal markers, with 26% of those neurons expressing TH+ positivity, confirming a dopaminergic phenotype. While these findings are limited to an in vitro model, they highlight the potential of Muse cells to differentiate into neurotransmitter-specific neuronal subtypes. This finding is of particular importance to PD, which is defined by the specific loss of dopaminergic neurons. Given their stress tolerance, targeted homing ability, and capacity for dopaminergic differentiation [133,141,142], Muse cells may be a promising candidate for cell therapy in PD.

As a defined subpopulation of MSCs, Muse cells appear to retain key immunomodulatory and neuroprotective properties, including modulation of microglial polarization, attenuation of TLR4-mediated inflammatory signaling, and secretion of trophic factors that support neuronal survival, synaptic plasticity, and neural repair [143,144]. Muse cells have also been shown to enhance regulatory T- cell activity, thereby promoting immune tolerance and dampening chronic inflammatory responses [145]. Through combined regenerative and immunomodulatory mechanisms, Muse cells may provide a dual therapeutic mechanism that is suited to ameliorate the complex pathophysiology of PD, where both neuronal loss and chronic neuroinflammation play critical roles in disease propagation.

### 4.2. Neural Stem Cells

Following transplantation, most NSCs remain undifferentiated. However, a small subset can adopt non-glial fates, including dopaminergic neurons, depending on their local environment. Even though less than 5% of transplanted NSCs adopt a dopaminergic phenotype [146], non-human PD models still exhibit meaningful functional and motor improvements, accompanied by more robust TH+ fiber staining [147]. These findings suggest that even limited neuronal differentiation may contribute to modest regenerative potential. Supporting this notion, Mine et al. grafted mesencephalic neuroepithelial stem cells, precursor of NSCs, into the rat striatum, resulting in increased number of TH+ cells and evidence of synaptic integration with reconstruction of host dopaminergic circuitry [148].

Despite these observations of regenerative potential, the predominant therapeutic mechanism for NSCs appears to be paracrine in nature [149]. By preferentially differentiating into astrocytes, NSCs secrete a wide array of neurotrophic factors, such as BDNF, IGF-1, NGF, and GDNF, to support neuronal survival and synaptic maintenance [150,151]. They also exert anti-inflammatory effects by reducing pro-inflammatory cytokines like TNF-α, IL-1β, and IL-2, while increasing anti-inflammatory cytokines, such as IL-10 [152,153]. In addition, NSCs promote anti-apoptotic signaling through the activation of the PI3k/Akt pathway, enhancing dopaminergic neuronal survival by mitigating oxidative stress and inhibiting programmed cell death [149]. Additional studies suggest that NSCs can reduce α-syn aggregation [154], further supporting host neuronal preservation.

While the aforementioned benefits are promising, sourcing of NSCs is ethically controversial as they are often derived from fetal tissue or embryonic stem cells. Use of these cells is also challenging due to the need for specific targeting and potential for immunogenicity during allogeneic transplantation. To overcome some of these challenges, investigators have explored human parthenogenetic derived NSCs (hpNSCs) as an alternative source. These cells are derived from unfertilized oocytes, which can generate an unlimited supply of cells. They also have reduced immunogenicity due to high HLA-G expression [155], making them more suitable for allogeneic transplantation. Although their in vivo differentiation capacity remains limited and their primary mechanism of action is largely paracrine like NSCs [156], hpNSCs have advanced into early-phase human clinical testing as a potential therapy for PD [157,158].

IC implantation of NSCs has shown potential therapeutic benefit, in large part due to local paracrine activity. Unlike other restorative therapies, however, systemic administration of NSCs has generally produced poor results. Despite smaller case series showing potential benefit of systemic routes [85,86], Lundberg et al. showed that neither IA nor IV administration of NSCs resulted in meaningful engraftment, likely reflecting insufficient chemotactic cues that impair accurate homing to distant brain regions [159]. IN delivery has been explored as a less invasive alternative, based on evidence of migratory ability [160] and successful neuronal differentiation in Alzheimer’s disease models [161]. In a phase I safety study in PD, IN-delivered NSCs produced modest improvements in motor scores in all subjects, though these benefits did not correlate with a sustained increase in dopaminergic uptake on imaging [92]. This incongruence suggests that IN delivery is insufficient to support the same meaningful regenerative effects seen in preclinical NSC implantation models. While IN delivery appears capable of supporting limited restorative effects, the widespread distribution of cells throughout the brain may restrict the number of cells that can reach target regions or the magnitude of local paracrine signaling needed to produce changes on functional imaging. Collectively, these findings highlight the need for larger clinical trials that can determine whether IN delivery can achieve sufficient CNS targeting to generate durable and meaningful therapeutic benefits.

## 5. Current Obstacles and Advancements in Regenerative Therapies

While dopaminergic cell replacement therapy offers a notable strategy to replenish diminishing stores of dopamine, significant obstacles revealed in pre-clinical studies, case reports, and clinical trials have limited its widespread adoption. The potential safety concerns with use of these cell types necessitate further product optimization and continued investigation with randomized studies.

### 5.1. Graft Induced Dyskinesias from Serotonergic Excess

One major disadvantage of FVM grafts is GIDs, which typically emerge 2–3 years post-transplantation, are unresponsive to complete cessation of dopaminergic therapies, and affect up to 50% of patients [22,162]. A leading hypothesis proposes that GIDs result from excessive serotonergic innervation that triggers an amphetamine-like release of dopamine from serotonergic terminals [163,164]. This theory was supported by the partial reduction in GIDs after the administration of buspirone, a 5-HT_1A_ receptor agonist that activates inhibitory serotonin autoreceptors [165]. Since FVM tissue inherently contains both dopaminergic and serotonergic neurons [166], the risk of GIDs is intrinsic to this tissue source. Efforts to minimize serotonergic contamination through careful dissection or immunoselection have been proposed to partially mitigate this risk [167,168]. In contrast, ESC- and iPSC-derived grafts undergo stringent in vitro patterning to create A9 dopaminergic neurons, with additional sorting to remove residual non-dopaminergic neurons [169], resulting in markedly lower rates of GIDs in both human and non-human studies [43].

### 5.2. Lack of Clinical Benefit Due to Batch Heterogeneity and Patient Selection

To date, the only regenerative stem cell therapies for PD tested in double-blind, randomized, placebo-controlled trials are FVM grafts [20,22,24]. Unfortunately, these studies demonstrated limited clinical efficacy, largely attributed to high biological variability of the grafts and the inclusion of participants with advanced-stage PD. Because fetal tissue varied substantially across donors, outcomes were inconsistent even within the same clinical center [67]. In contrast, ESC- and iPSC-derived dopaminergic progenitors are produced under good manufacturing practice conditions, which markedly reduce graft heterogeneity. While some degree of biological variability remains because of differences in donor cell lines and differentiation efficiencies, the variability is substantially lower than that observed with FVMs because of newer standardized differentiation and cell-sorting protocols [53,54,55,56]. Current studies of ESCs and iPSCs are unblinded with limited follow up (≤24 months), preventing evaluation of long-term efficacy in light of these advancements. While preliminary results from small, unblinded cohorts appear promising, caution is warranted given the potential for expectancy bias.

Patient selection for trials is also an important consideration, as early and advanced PD differ in the degree of nigrostriatal degeneration, quantity of α-synuclein aggregates, and level of responsiveness to dopaminergic therapy [170,171,172]. Advancing disease is associated with substantial neuroinflammation. This process results in synaptic reorganization [173] and disruption of dopamine and other neurotransmitter circuits [174]. Post hoc analyses of FVM trials reflected these differences, revealing that patients with milder disease (UPDRS ≤ 49) [22] or robust L-dopa responsiveness (≥50%) [175] had significantly better outcomes. Similarly, younger patients (<60 years) [20] demonstrated greater graft integration and functional benefit, supporting the influence of age-related impairment in neurogenesis, by way of endogenous repair and release of neurotrophic growth factors [176].

### 5.3. Decreased Cell Survival Due to Unfavorable Host Microenvironment

The low survival rate of transplanted cells remains a challenge for the field, as estimates suggest that at least 90% of transplanted neurons die within the first week post-transplantation [177]. Two critical areas of vulnerability include the in vitro differentiation phase and the peri-implantation phase [178]. During the differentiation process, grafts are placed in environments that result in mechanical stress from direct cell-to-cell contact, cell dissociation, and toxic patterning chemicals that trigger apoptosis [53,179,180,181]. During the implantation step, a needle is inserted into the brain parenchyma which triggers localized inflammation resulting in the release of pro-inflammatory cytokines and microglial activation [182]. This inflammation adds to an already toxic environment, marked by reduced neurotrophic support and impaired endogenous neurogenesis, thereby compromising graft survival [183]. Given the hostile host environment and intrinsic vulnerability of dopaminergic neurons under stress [184], meaningful graft survival and functional integration of transplanted cells will require strategies that improve cell viability during differentiation and sustain survival after implantation.

Several strategies have been explored to decrease cell losses. Kim et al. discovered that one could optimize in vitro patterning in a 3D organoid culture to reduce mitochondrial stress, enhance maturation, and improve viability [185]. Increasing the interval between needle insertion and cell delivery by at least 1 h has been shown to reduce injection-induced inflammation that results in cell destruction [186]. Co-administration with substances such as caspase inhibitors, calcium channel blockers, or lipid peroxidation inhibitors have shown to improve overall graft survival through the inhibition of apoptosis [187,188,189]. Additional strategies to decrease immediate post-transplantation inflammation include co-transplantation with regulatory T cells [182] or astrocytes [190], which enhances engraftment through immunomodulatory and trophic support. For instance, Song et al. demonstrated that astrocyte co-engraftment can reduce α-syn pathology in the brain [191]. Similarly, pre-treating the graft medium with growth factors such as GDNF, BDNF, bFGF, or IGF-1 promotes synaptic integration by strengthening axonal connections [192,193,194,195], further enhancing survival rates and functional recovery.

More recently, the integration of nanotechnology into cell therapy has emerged as a viable solution to several of these challenges. Through the manipulation of nanoscale materials, researchers have created new structures and scaffolding with improved functionality to augment the effects of stem cells. Nanomaterials such as collagen hydrogels, self-assembling peptides, and poly(lactic-co-glycolic acid) scaffolds, can function as controlled delivery systems for trophic molecules and graft protection to improve their survival [196,197,198,199]. They can also improve the regeneration capacity of the grafts by shielding them from destructive forces in their environment over time [200]. Results of these studies demonstrate superior efficacy compared to co-implantation with the neurotrophic factor alone, likely due to sustained trophic support afforded to stem cells encapsulated in these carriers [201].

Additionally, nanoscale scaffolds can promote stem cell differentiation into dopaminergic cells, while nanopatterning facilitates precise migration to specific areas [202]. Xu et al. used injectable hydrogels containing tannic acid and gold nano-crosslinkers to improve NSC proliferation and dopaminergic differentiation in rat models [203]. Self-assembling peptide scaffolds, like RADA16-I, improve viability and differentiation efficiency by creating an extracellular matrix-like niche to increase cell survival by over 100-fold, compared to regular cell suspension [204]. Polymetric nanoparticles loaded with perfluoro-1, 5-crown ether and complex microRNA-124 allow for modulation of gene expression of the stem cells to strengthen specific neuronal lineage commitment [205]. Finally, magnetic nanoparticles utilize external magnetic fields to promote precise delivery and regional enrichment of grafted stem cells, which is of particular relevance for poor migratory cell subtypes [206].

### 5.4. Accumulation of Alpha Synuclein Pathology in Grafted Cells

Implantation of stem cells into a diseased microenvironment not only limits their survival, but it also subjects them to the ongoing disease process. Postmortem studies of grafted patients 10–24 years after FVM transplantation reveal preserved, robust dopaminergic innervation, but also the gradual accumulation of α-syn [207]. These deposits can appear as early as 4 years post-transplantation [208], yet the effects of the graft itself start to wane about 12–14 years post-transplantation [209]. It is estimated that about 11–30% of the fully matured implanted grafts develop some degree of α-syn build-up, often expressing little to no TH+ immunofluorescence over time [210,211]. These findings reveal that implanted cells are susceptible to the host’s pathological environment, acquiring Lewy body pathology and losing dopaminergic function over time [212].

Although patients with ESC and iPSC grafts have not yet been followed long enough to observe such effects, it is likely that they will exhibit similar vulnerabilities given their primary role of dopamine replacement rather than modulation of the inflammatory milieu. Future strategies can prioritize therapies or treatment plans that provide both dopaminergic replacement and immunomodulatory benefits, such as more research in the feasibility of NSCs, hpNSCs, and Muse cells as a treatment option in PD. Alternatively, one could combine IC cell replacement with periodic, less invasive adjunctive cell therapies to support graft viability and mitigate progressive host pathology.

## 6. Current Obstacles and Advancements in Restorative Therapies

Compared to regenerative therapies, restorative therapies produce less robust and sustained improvement in clinical symptoms [44]. Many of the practical and biological limitations discussed below are better illustrated with MSC-based approaches, given the administration route limitation with NSCs.

### 6.1. Decreased Cell Survivability

One mechanism by which MSCs exert neuroprotective effects is by modulating microglia towards a resting state after engulfment. To maximize their immunomodulatory effects, MSCs have to remain viable long enough to release trophic factors before phagocytosis occurs. However, the rapid decline in MSC numbers soon after administration remains a major challenge. Low survival rates have been reported in direct transplantation [213], and IV delivery leads to an estimated 80–90% of cells becoming trapped in the pulmonary vasculature because of their size [214]. Once trapped, MSCs initially remain viable and secrete trophic factors; however, shear forces, anoikis, and exposure to the inflammatory environment rapidly reduce their numbers [214,215,216]. Notably, this clearance is not entirely detrimental, as MSC engulfment also contributes to downstream immunomodulatory effects, as discussed above.

Given these challenges, efforts have focused on strengthening early survival and functional activity of the secretome. Conditioning MSCs with α-syn or co-culturing with inflammatory markers, such as IFNγ, TNFα, or IL-1β, enhances secondary immunomodulatory activity by increasing susceptibility to apoptosis [217,218]. Pre-treatment through hypoxic conditioning, antioxidant supplementation (e.g., vitamin E, melatonin), or genetic modification (e.g., HIF1α) have been shown in various neurological disease models to promote gene stability, enhance paracrine function, and improve cell viability in harsh microenvironments [219,220,221,222]. Furthermore, strategies to improve MSC biodistribution have been explored via incubation in hyperosmolar solutions, which reduces cell size while preserving mitochondrial and DNA integrity [223]. This reduction in size allows for a portion of MSCs to bypass pulmonary entrapment and reduce the mechanical stress incurred during travel. While these findings were demonstrated in immunocompetent mice, they suggest potential approaches for use in neurodegenerative diseases.

### 6.2. Necessity of Repeated Dosing

Given the transient nature of MSC-mediated effects and the progressive neurodegeneration in PD, there is an inherent need for repeated administration for long-lasting benefits. This limitation also illustrates the importance of establishing efficacy through minimally invasive delivery methods, as repeated IC transplantation is neither safe nor practical.

Shigematsu et al. investigated 5 or 6 repeated IV doses of autologous Ad-MSCs in 3 non-blinded patients, given at 1 month intervals [90]. Although clinical improvements were observed, the benefits appeared to diminish after the third infusion, suggesting a potential ceiling effect of MSC-mediated neural repair. Following an initial successful pilot study in a single patient receiving multiple autologous IV Ad-MSC [91], Vij et al. administered 6 repeated IV infusions of Ad-MSCs every 6 weeks in 10 subjects, observing modest clinically meaningful improvements in motor function at 26 weeks, though not statistically significant [94]. However, the absence of biomarker or imaging assessments in these studies limited interpretation of restorative effects. These initial positive findings have supported the initiation of multiple ongoing phase I and II trials evaluating repeat IV Ad-MSC dosing in PD [224].

Collectively, these studies suggest that repeated dosing may improve or prolong clinical benefit. There may be a saturation threshold beyond which additional cell delivery does not yield additional benefit, but larger controlled trials are needed to clarify the optimal number and interval of MSC administration in PD. Furthermore, while IV is the most commonly used route of administration, additional investigation into other minimally invasive approaches may be warranted to facilitate safe and practical repeated dosing strategies.

### 6.3. Decreased Penetrance of the Blood–Brain Barrier

The most optimal administration route for MSCs in PD remains unclear, because of heterogeneity in delivery methods, tissue sources, and cell preparation techniques. What is clear, however, is that the use of IV delivery results in limited penetrance of MSCs and their trophic factors across the BBB [225]. To address this limitation, MRI-guided focused ultrasound has been utilized to temporarily increase BBB permeability and enhance transmigration of the MSCs and MSC-derived growth factors into the brain [226]. This alteration allows for an increase in TH+ cell density after IV administration, likely mediated by the upregulation of chemokines and cytokines that promote homing and neuroprotection. Furthermore, this method has been shown to be well tolerated in animal PD models [227]. Additionally, to improve MSC brain targeting, dextran-coated iron nanoparticles have been employed. These particles enhance MSC migration toward damaged sites and improve neuroprotective effects on surviving dopaminergic neurons [228]. While in vitro studies suggest that this technology may also improve the dopaminergic differentiation potential of MSCs, it is unknown if these efforts can be reliably reproduced in vivo PD models [229].

## 7. Conclusions

Cell therapies represent a potentially beneficial strategy for the treatment of PD, with the potential to provide disease modifying benefit. A clearer understanding of the distinct biological roles that different stem cells play is essential for appropriately framing therapeutic expectations and interpreting clinical outcomes. As outlined above, cell types such as FVM cells, ESCs, and iPSCs, primarily possess regenerative potential, whereas MSCs display predominantly restorative effects. Muse cells and NSCs fall along an intermediate position, representing hybrid therapeutic approaches. The delivery route of these cells further influences their efficacy and plays an important role in their position along the regenerative-restorative continuum. Since the framework is intended to provide a conceptual perspective rather than a comprehensive assessment of the literature, a systematic search was not utilized. While efforts were made to synthesize key preclinical and clinical studies in the field, there might be publication and selection bias present.

When considered together, the respective advantages and limitations of these approaches suggest that regenerative and restorative strategies may be complementary rather than mutually exclusive. For example, poor graft survival and progressive accumulation of α-syn within transplanted regenerative cells may be ameliorated by the ongoing neuromodulatory and anti-inflammatory effects provided by restorative therapies. Such an integrated approach might result in more durable clinical benefit compared to either strategy alone [75,88]. However, this hypothesis is preliminary. Before combined or sequential strategies can be meaningfully tested, individual cell types and modifications must be evaluated independently using rigorously designed studies to establish reproducible efficacy and safety profiles. Currently, many ongoing clinical trials involve small cohorts and unblinded designs, limiting the ability to draw definitive conclusions regarding comparative effectiveness or optimal implementation.

Substantial challenges remain, particularly with respect to variability in stem cell sourcing, processing, manufacturing, and treatment protocols. Addressing this heterogeneity is a critical prerequisite before more complex translational questions can be systematically evaluated, including optimal delivery routes, dosing schedules, and the sequential versus simultaneous administration of different cell therapies. For example, approaches utilizing intracerebral delivery introduce further logistical and ethical considerations due to their invasive nature, despite the potential to confer greater therapeutic efficacy. This may limit patient acceptance and pose recruitment challenges, highlighting the difficulty of robustly evaluating the theoretical advantages of such approaches in larger confirmatory trials. Longer term follow-up is also needed, particularly for MSC-based therapies, to better characterize durability of effect, survivability, and potentially delayed adverse outcomes. Given these uncertainties, specific clinical trial designs or definitive therapeutic recommendations cannot yet be accurately proposed. Continued investigation and standardization will be essential to inform future translational strategies.

## Figures and Tables

**Table 1:** List of Completed Clinical Studies in Parkinson’s Disease Using Regenerative Therapies.

First Author (Year)	Stem Cell Type	Study Design	Sample Size	Follow-Up (Months)	Age ^a^	Disease Duration (Years) ^a^	Clinical Outcomes	Safety/Adverse Events
**Lindvall et al.** ^b^ **(1989)** [6]	FVM	case series	2	6	48 and 55	15	Minor motor improvement; no changes in daily ON time	Fever
**Hitchcock et al. (1990)** [7]	FVM	case series	12	12	55.8	16	Clinical motor improvement; decrease in LEDD and dyskinesias	NR
**Madrazo et al. (1990)** [8]	FVM	case series	4	11	48.5 ± 3.1	11.8 ± 3.4	Motor and QoL improvement	Bone flap infection, brain abscess, thrombophlebitis
**Henderson et al. (1991)** [9]	FVM	case series	12	12	56 ± 6.2	18	Varied UPDRS changes (improvement vs. unchanged vs. deterioration)	Depression
**Freed et al.** ^c^ **(1992)** [10]	FVM	case series	7	11–46	56.3 ± 9.3	16.2 ± 10	UPDRS and QoL improvement; decreased motor fluctuations; reduced dyskinesias	NR
**Widner et al. (1992)** [11]	FVM	case series	2	24	43 and 30	10	Total UPDRS improvement; reduced dyskinesias	Bronchitis
**Molina et al. (1993)** [12]	FVM	case series	5	3	51	7–14	Significant clinical improvement; reduced LEDD	NR
**Peschanski et al. (1994)** [13]	FVM	case series	2	10 and 17	63 and 49	17 and 10	Motor improvement; decreased motor fluctuations; reduced dyskinesias	OCD, depression, anxiety, euphoria, UTI
**Freeman et al. (1995)** [14]	FVM	case series	4	6	52.25 ± 10	14.25 ± 6	Improvement in total UPDRS; increase in ON time	Superficial cortical hemorrhage, confusion, hallucinations
**Defer et al. (1996)** [15]	FVM	case series	5	15–36	57.4 ± 8	17.2 ± 3	Total and motor UPDRS improvement	GID, transient frontal lobe syndrome
**Kopyov et al. (1996)** [16]	FVM	case series	22	6–24	55.2	NR	Improvement in UPDRS, ADL and dyskinesia scores; 4 non-responders	NR
**Levivier et al. (1997)** [17]	FVM	case series	3	12	NR	NR	UPDRS improvement; increased daily ON time	Cushing syndrome, confusion, dyskinesias
**Hauser et al. (1999)** [18]	FVM	case series	6	24	55.5 ± 9.3	18.2 ± 7.6	Improvement in UPDRS and ADL scores; increase in ON time; reduced dyskinesias	Cortical hemorrhage, nausea and dehydration
**Brundin et al. (2000)** [19]	FVM	case series	5	18–24	53 ± 9.8	12.6 years	Improvement in UPDRS motor score, decrease in OFF time; 1 non-responder	Peripheral thrombophlebitis, nocturnal confusion, depression, personality change, apathy
**Freed et al. (2001)** [20]	FVM	Phase II	40	12	57 ± 10	14 ± 6	No significant difference in UPDRS in whole cohort; significant improvement of UPDRS in younger patients	No significant difference in severity of AE between groups
**Mendez et al. (2002)** [21]	FVM	case series	3	13	53 ± 5.6	11.7 ± 2	Improvement in motor UPDRS and ADL scores in OFF; variable change in LEDD	Cerebral hemorrhage
**Olanow et al. (2003)** [22]	FVM	Phase II	34	24	58.5 ± 8.4	10.9	No significant difference in UPDRS in whole cohort; significant improvement of UPDRS in milder patients	More frequent AEs in transplantation group; GID
**Schweitzer et al. (2020)** [23]	iPSC	case report	1	24	69	10	Motor and QoL improvement	NR
**Barker et al. (2025)** [24]	FVM	Phase II	27	36	51.8 ± 9.2 (transplantation); 54.6 ± 4.7 (control)	NR	No significant difference in UPDRS in whole cohort; decreased OFF time in transplant group	More frequent AEs in transplantation group; GID
**Chang et al. (2025)** [25]	ESC	phase I/IIa	12	12	60.3 ± 5	10.5 ± 2.5	Dose dependent improvement in OFF MDS-UPDRS III and H&Y scores; decrease in daily OFF	Cerebral hemorrhage, new onset DM, idiopathic thrombocytopenia; no product related AEs
**Sawamoto et al. (2025**) [26]	iPSC	phase I/II trial	7	24	NR	NR	Improvement in MDS-UPDRS and H&Y; no change in PDQ-39	No difference in severity and amount of AE based on dosage; dyskinesias
**Tabar et al. (2025)** [27]	ESC	phase I	12	18	67 (64.5, 70)	9 (5.9, 11.5)	Dose dependent improvement in OFF MDS-UPDRS III and ON time	COVID, seizure, GI hemorrhage; no product related AEs

Abbreviations: **ADL**: activities of daily living; **AE**: adverse event; **ESC**: embryonic stem cells; **FVM**: fetal ventral mesencephalic cells; **GI**: gastrointestinal; **GID**: graft induced dyskinesia; **H&Y**: Hoehn and Yahr rating scale; **iPSC**: induced pluripotent stem cells; **LEDD**: levodopa equivalent daily dosing; **MDS-UPDRS**: Movement Disorders Society—Unified Parkinson’s Disease Rating Scale; **NR**: not reported; **OCD**: obsessive compulsive disorder; **PDQ-39**: Parkinson’s disease questionnaire—39; **UPDRS**: Unified Parkinson’s Disease Rating Scale; **UTI**: urinary tract infection. ^a^ The values are given as mean ± SD, median (Q1, Q3), or ranges, depending on the information that is given in the articles. ^b^ This article represents the initial report from the Lund transplantation cohort. Several subsequent publications from the same group provide longer-term clinical follow up of overlapping implanted patients, with the longest assessments extending 3 years post implantation. These studies reported more robust increases from baseline in UPDRS, reduction in time spent in the OFF state, and a reduction in LEDD [28,29,30,31]. ^c^ This article represents the final comprehensive report of the Freed transplantation cohort, incorporating outcomes from all enrolled publications. Earlier publications from this group reported shorter-term follow up, while subsequent studies reanalyzed this cohort, examined staged bilateral implantation strategies, or extended the longitudinal follow-up [32,33,34,35].

## Data Availability

No new data were created or analyzed in this study. Data sharing is not applicable to this article.

## Abbreviations

The following abbreviations are used in this manuscript:

6-OHDA	6-hydroxydopamine
Ad-MSC	adipose mesenchymal stem cell
α-syn	alpha synuclein
BBB	blood–brain barrier
BM-MSC	bone marrow mesenchymal stem cell
ESC	embryonic stem cell
FVM	fetal ventral mesencephalic
GID	graft induced dyskinesia
HIE	hypoxic ischemic encephalopathy
IA	intra-arterial
IC	intracerebral
ICH	intracerebral hemorrhage
IN	Intranasal
iPSC	induced pluripotent stem cell
IT	intrathecal
IV	intravenous
MSC	mesenchymal stem cell
Muse	multilineage differentiating stress-enduring
NSC	neural stem cell
PD	Parkinson’s Disease
ROS	reactive oxygen species
SDF-1α	stromal- cell derived factor 1α
SNpc	substantia nigra pars compacta
TH	tyrosine hydroxylase
UC-MSC	umbilical cord derived mesenchymal stem cell
UPDRS	Unified Parkinson’s Disease Rating Scale
